# Insertionally polymorphic sites of human endogenous retrovirus-K (HML-2) with long target site duplications

**DOI:** 10.1186/s12864-017-3872-6

**Published:** 2017-06-27

**Authors:** Tomoaki Kahyo, Hidetaka Yamada, Hong Tao, Nobuya Kurabe, Haruhiko Sugimura

**Affiliations:** Department of Tumor Pathology, Hamamatsu University School of Medicine, Hamamatsu, Shizuoka Japan

**Keywords:** Human endogenous retrovirus, Human genome database, Insertional polymorphism, Target site duplication

## Abstract

**Background:**

Human endogenous retroviruses (HERVs) belong to the LTR-retrotransposon family, where the complete HERV sequence contains two long terminal repeats (LTRs) located at each end. Intact LTRs possess highly conserved transcriptional promoter and enhancer sequences, so analyses of HERV insertional polymorphisms are expected to provide greater insights into human genomic variation compared with the conventional analysis of single nucleotide variations. High-throughput sequencing technology is developing but genome-wide investigations of HERVs are methodically challenging, and thus a comprehensive understanding of HERV insertional polymorphisms and target site duplications (TSDs) remains elusive.

**Results:**

We identified five human-specific insertionally polymorphic sites in HERVK (HML-2), one of the HERV subgroups, by extracting HML-2-deleted sequences from the genomic structural variation database, which we successfully characterized and then updated the existing catalogue of HML-2 insertional polymorphisms. The insertionally polymorphic states were confirmed in a small Japanese population by genomic PCR analysis for four of the five sites identified. Sequencing of the preintegration sites clearly showed that the HML-2 site located at 7p21.2 had 250-base pair (bp) TSDs, which is one of the longest TSDs in HML-2. In addition to these five sites, another insertionally polymorphic site for a non-human-specific HML-2 site was also identified at 6p25.2, which was flanked by 111-bp TSDs and the corresponding ERV locus was also annotated in the genome of non-human primates.

**Conclusions:**

Our analysis demonstrated the existence of HERV insertions flanked by unconventionally long TSDs, including those with lengths as high as 250 bp. This suggests that the length range of retroviral TSDs is larger than considered previously, which might help to understand how retroviral integration occurs in the host genome.

**Electronic supplementary material:**

The online version of this article (doi:10.1186/s12864-017-3872-6) contains supplementary material, which is available to authorized users.

## Background

HERV-K (HML-2) is a subgroup of human endogenous retroviruses (HERVs), which are considered to be the most recently acquired members of the HERV family, and most human-specific or insertionally polymorphic HERVs belong to this group [1, 2]. A retroviral provirus comprises double-stranded DNA integrated in the host genome, with long terminal repeats (LTRs) at each end and several open reading frames (ORFs) between the two LTRs. Homologous recombination between the two LTRs is known to yield a solo LTR (sLTR). The two elements located in 19p12b and Xq21.33, where the former is known as K113, are intact proviruses in terms of all viral proteins and most HML-2 loci are present as a sLTR [3, 4]. The physiological and pathogenic effects of insertional HML-2 variants are not yet understood [1].

Insertions and deletions (indels) of long sequences are technically difficult to detect compared with single nucleotide variations and a comprehensive understanding of indels remains elusive. In particular, an overall understanding of insertional polymorphisms in HERVs is not available at present due to the large sizes of the sequences (full length ~ 10 kbp) and the large number of copies (>200,000 loci), collectively accounting for about 8% of the human genome [1]. Retroviral invasions of the host genome are believed to have occurred in the germ lines of humans and human ancestors, and the retroviral elements were passed on to modern humans as a heritable part of the genome. Target site duplications (TSDs) typically comprises 5- or 6-base pairs (bp) in HERV-K elements and they are formed during retroviral integration into the host genome. Insertional polymorphisms in HERVs are assigned according to the presence or absence of a specific HERV sequence. The existence of human-specific insertional polymorphisms in HERVs implies that exogenous retroviral integration occurred within the genome of an ancestor’s germline cells after the divergence of humans from non-human primates [5].

Recently, high-throughput sequencing technologies have provided the means to identify insertionally polymorphic sites in HERV-K in several investigations [3, 6, 7]. In these studies, the Illumina whole-genome sequencing (WGS) data obtained from the Cancer Genome Atlas Project, EGS500 project, 1000 Genomes Project, or Human Genome Diversity Project were analyzed using Tea or RetroSeq software to identify LTR-supporting read pairs [3, 6, 7]. It is not easy to identify ERVs in repeat regions by mining WGS data because it is necessary to ensure sufficient coverage of the genome and it is possible that WGS reads might not be mapped to one specific location or that ERVs may be present within larger encompassing variants. Therefore, although 40 HML-2 loci have been assigned as insertional polymorphisms in the human genome in recent decades (Additional file 1: Table S1), the overall abundance of HML-2 insertional polymorphisms is still unknown. In this study, we investigated the insertionally polymorphic states of HML-2 sites in the human hg19 reference genome using the Database of Genomic Variants (DGV), which is a curated catalog of human genomic structural variations in healthy control samples, and we clearly demonstrated the existence of retroviral elements with long TSD sequences, including those measuring up to 250 bp in length. Our observations suggest that retroviral insertions can produce longer TSDs than considered previously. In addition, we demonstrated the existence of a non-human primate ERV locus corresponding to an insertionally polymorphic HML-2 with long TSDs, thereby suggesting an ancestral polymorphism or homologous recombination between the two long TSDs.

## Methods

### Data analysis

**Fig. 1:**
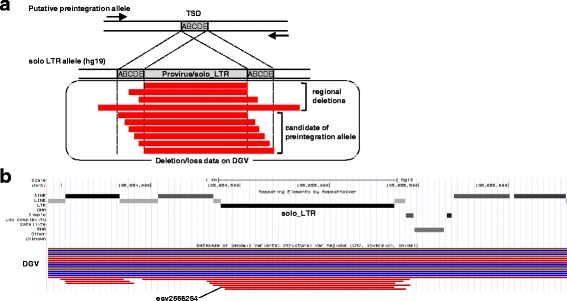
Extraction of HML-2 insertional polymorphisms. **a** Scheme for estimating HML-2 insertional polymorphisms. Deletion and loss variant types were selected from the DGV and collated using the hg19 reference genome data. The primers used for genomic PCR are shown by *arrows*. *Letters* indicate nucleotides in the TSDs. **b** Display of the UCSC Genome Browser around the region of 195,654,396–195,655,363 on chromosome 3. *Red* and *blue bars* denote gain and loss regions, respectively. The deletional variant of esv2668264 indicates the presence of a preintegration allele at this LTR site. The other deletional forms in the area displayed could not be assigned strictly as preintegration alleles

The GRCh37/hg19 reference genome was used as the reference for all the genomic positions. RepeatMasker and structural variant data were downloaded from the University of California Santa Cruz (UCSC) (http://genome.ucsc.edu/index.html; data date, 2013–3-26; last accessed July 2016) and DGV (http://dgv.tcag.ca/dgv/app/home; GRCh37_hg19_variants_2015–07-23.txt, last accessed July 2016) archives, respectively [8, 9]. Source information for the DGV data is shown in Additional file 1: Table S2. To extract putative insertionally polymorphic sites from the HML-2_LTRs, DGV data were first analyzed by a custom-made Perl script, which is available via GitHub (https://github.com/TKahyo/IvDd.pl), with the following filters: (i) DGV variant subtype “loss” or “deletion”; (ii) deletional regions covering the HML-2_sLTR sequence; and (iii) length of the deletional region shorter than 1500 nucleotides. As a result, we identified 21 candidates, which ranged from 364 to 975 bp. In the case of a provirus, the difference between the length of the DGV deletion and that of the partial proviral region ranging from one side of the LTR to the ORFs was applied to the length filter described above in (iii). Next, among the filtered data, the deletion site that was consistent with the preintegration status was selected manually using the UCSC Genome Browser (Fig. 1a). It should be noted that it was necessary to coordinate the start positions from the DGV deletion data as well as RepeatMasker. The hg19 axtNet data were compared against panTro4, gorGor3, and ponAbe2 via the UCSC for primate genomes.

To calculate the putative TSD lengths, sequences flanking the HML-2 sites were extracted from the reference genome data using a custom script, which is available via GitHub (https://github.com/TKahyo/LTSD.pl), and based on the following algorithm: (i) extract the sequences on the left and right flanks of LTRs in the 4–500 bp range; (ii) globally align two flanking sequences using the sequence comparison algorithm T-Coffee [10]; (iii) select a pair of longest sequences as putative TSDs with high identity scores, where the identity ratio was greater than 0.75 and the number of mismatches and gaps (“N”) was no more than one (for a length < 10 bp), two (for a length < 20 bp), or three (for a length ≥ 20 bp). For proviruses, we also assessed whether a pair of LTRs were in the same orientation and whether the gaps between the LTRs and the nearest HERVK-int ranged from −1 (one base overlapping) to 100 bp. The lengths of the putative TSD sequences were calculated from the alignment of the selected pair.

### Specimens

The samples used for validating the public data archives were chosen randomly from a DNA collection of Japanese volunteers aged ≥60 years without cancer morbidity, which was obtained by Iwata City Hospital. [11]. There were 23 or 24 samples in total (Table 1).

**Table 1:** Candidates for novel HML-2 pre sites

Chr	Position	Region	Structure	ID	Genomes of other primates^a^	Frequency^b^			Total alleles	Age^c^ (MYA)	Reference^d^
						*pre*	*pro*	*solo*			
chr3	195654396–195655363	3q29	solo LTR	esv2668264	ins	0.152	0	0.848	46	2.27	[3]
chr4	120263689–120264654	4q26	solo LTR	esv2661724	ins	0.708	0	0.292	48	2.11	[3]
chr7	16237347–16238314	7p21.2	solo LTR	esv2662783	ins	0.5	0	0.5	48	2.42	this study
chr8	37050886–37051853	8p11.23	solo LTR	esv2677273	ins	0.813	0	0.187	48	2.15	this study
chr11	71875418–71876385	11q13.4	solo LTR	esv2675196	ins	n.d.	n.d.	n.d.	n.d.	2.35	[3]
chr6	3055034–3055749	6p25.2	LTR+ ORF + 7bp^e^	esv2731485	ins panTro4 /ponAbe2	0.435	0.565	0	46	n.d.	this study

^a^Reference genomes for chimpanzee (PanTro4), gorilla (gorGor3), orangutan (PonAbe2), and rhesus macaque (rheMac8). ins: Retroviral insertion

^b^Tested in this study; *pre*: preintegration, *pro*: provirus, *solo*: solo LTR, *n.d.* not determined

^c^The previous study by Subramanian et al. was used to obtain the ages of solo LTRs [12]. *MYA* million years ago, *n.d.* not determined

^d^Insertional polymorphisms were inferred from the previous study by Wildschutte et al. [3].

^e^The ORF was partially deleted and the flanking 7-bp sequence was assigned as part of the ORF

### Genomic PCR

Genomic DNA was extracted using a DNeasy Blood and Tissue Kit (Qiagen, Valencia, CA). The primers used for genomic PCR are listed in Additional file 1: Table S3. Pfu-x polymerase (Greiner Bio-One, Frickenhausen, Germany) was used for validating the insertional polymorphisms. After agarose gel electrophoresis, the amplified DNA was extracted and sequenced directly using a BigDye Terminator v3.1 Cycle Sequencing Kit (Applied Biosystems, Foster City, CA) and a capillary sequencer (3130 Model, Applied Biosystems).

## Results

### In silico analysis of HML-2 insertional polymorphisms based on the DGV

In this study, long indels covering HERV and TSD sequences were not assigned to the category of “HERV insertional polymorphisms,” but instead we designated them as “regional indels” (Additional file 2: Figure S1). HML-2-related LTRs (HML-2_LTRs) include members of LTR5 and LTR5_Hs, and they were classified based on their phylogeny and characteristic sequences, and annotated in the human reference genome using RepeatMasker via the UCSC website. This group also included indels reported as non-reference LTRs, as listed in Additional file 1: Table S1. The other HML-2-related LTRs, LTR5A and LTR5B, were not included in the HML-2_LTRs in this study because their integration into the host genome is considered to have occurred before the integration of LTR5_Hs [12]. The number of autosomal HML-2_LTRs (LTR5_Hs/LTR5) was inferred as 622 based on the RepeatMasker data. This number is still arguable because the inferred HML-2_LTRs included those measuring less than 50 bp and those located close to another LTR within 10 bp. Based on a survey of previous reports, a list was prepared of HML-2 insertional polymorphisms for which the allelic status could be defined as preintegration or insertion (Additional file 1: Table S1). This list shows that 40 HML-2 sites were present as insertional polymorphisms in the human genome. To investigate other insertional polymorphisms in the HML-2_LTRs, we extracted structurally variable HML-2 regions from the DGV database. We retrieved the DGV deletion data corresponding to an HML-2 element and one side of the flanking duplicated sequences (Fig. 1a and b). We also classified the extracted HML-2 insertional polymorphisms into human-specific and non-human-specific groups based on the genomes of non-human primates. Three of the candidate sites identified by our analysis were recently inferred by Wildschutte et al. [3], but their preintegration sites were not validated. Finally, after excluding the sites validated previously as insertional polymorphisms, five and one HML-2 sites were identified as candidate human-specific and non-human-specific insertional polymorphisms, respectively (Table 1).

### Genomic PCR for human-specific HML-2 insertional polymorphisms

**Fig. 2:**
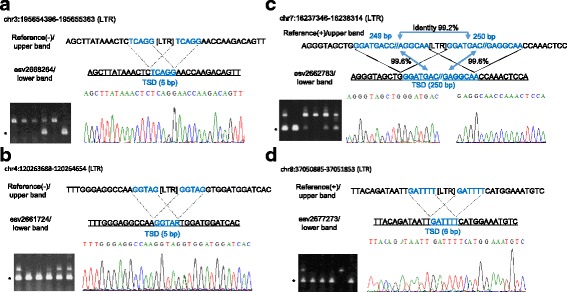
Sanger sequencing of preintegration alleles. Validation of insertional polymorphisms at chr3:195,654,396–195,655,363 (**a**), chr4:120,263,688–120,264,654 (**b**), chr7:16,237,346–16,238,314 (**c**), and chr8:37,050,885–37,051,853 (**d**) by Sanger sequencing. Fluorescence data for the *underlined* sequences are shown below. The TSD sequences are shown by *blue* characters. R is a G/A SNP. *Asterisks* show the PCR products containing a preintegration site

**Fig. 3:**
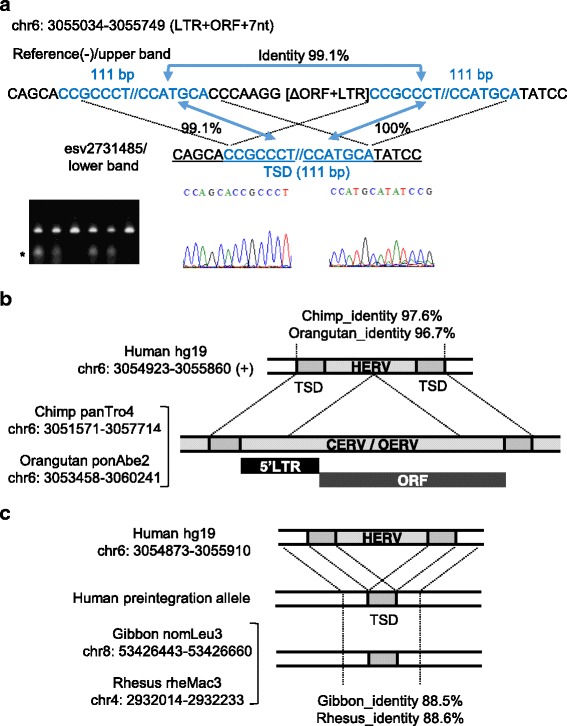
HML-2 structural variation at 6p25.2. **a** Validation of structural variations at chr6:3,055,034–3,055,749 using Sanger sequencing. Fluorescence data for the *underlined* sequences are shown below. *Blue* characters and *asterisks* show TSD sequences and the presence of preintegration alleles, respectively. **b** The LTR site at chr6:3,055,034–3,055,749 was an ortholog of ERVs in the chimpanzee (panTro4; chr6: 3,051,682–3,057,596) and orangutan (ponAbe2; chr6: 3,053,569–3,060,123) reference genomes. The regional positions indicated include TSDs. **c** Putative preintegration alleles corresponding to the LTR site at chr6:3,055,034–3,055,749 in hg19 were assigned in the gibbon (nomLeu3) and rhesus macaque (rheMac3) reference genomes. The regional positions indicated include TSDs and the surrounding regions. *Gray* regions indicate TSDs. CERV and OERV denote ERV elements in the chimpanzee and orangutan, respectively

Genomic PCR and direct sequencing of the PCR products were conducted to validate the insertionally variable states of the extracted human-specific HML-2 sites. The results confirm insertionally polymorphism for five of the HML-2 sites, also demonstrating the TSD presence (Figs. 2 and 3). ORFs were found in the 6p25.2 locus, but the other four HML-2 loci were solo LTRs based on the reference genome GRCh37/hg19, and no proviral alleles were detected in the four HML-2 loci in the test population. The allele frequencies of the preintegration state (HML-2_pre) ranged from 0.152 to 0.813 in the Japanese test population (*n* = 23 or 24) (Table 1). These results show that the five HML-2 loci at 3q29, 4q26, 8p11.23, 7p21.2, and 6p25.2 are insertionally polymorphic and they appear to be present as common variants, at least in the Japanese population. The other human-specific HML-2 locus at 11q13.4, which was also a solo LTR, was not amplified under our PCR conditions. It would be very difficult to specifically amplify this region because this locus is encompassed by multiple repetitive sequences such as L1 and Alu (Additional file 3: Figure S2 and Additional file 1: Table S3).

### Long TSD sequence at the 7p21.2 LTR site

The sequence of a TSD could be clearly determined from the HERV insertion and preintegration sequence information. In particular, the TSD sequence at 7p21.2, which was 99.6% identical to the LTR-flanking duplicated sequence on either side, measured 250 bp in length (Fig. 2). The presence of the long LTR-flanking duplicated sequences at 7p21.2 was shown in a previous study by Mamedov et al., where the HML-2 locus was compared with the preintegration sites in other primates, but the presence of the preintegration allele at 7p21.2 in the human genome was unknown [13]. Therefore, our finding of the preintegration allele at 7p21.2 is important because the existence of the long TSD sequence was clearly validated. The putative HML-2 TSD sequences and the distribution of their lengths were inferred by computational analysis of our estimated 622 LTR5_Hs sites in the range of 4–500 bp. In total, we estimated the presence of 451 putative TSD sequences for HML-2_LTR sites (Additional file 4: Figure S3). The TSD sequence at 7p21.2 was the longest among the known HML-2 insertional variants and the second longest among all HML-2 sites (Table 2, Additional file 1: Table S4, and Additional file 5: Figure S4). This indicates that retroviral integration into the host genome region corresponding to human 7p21.2 yielded an unusually long TSD.

**Table 2:** Estimated long duplicated sequences (>10 bp) flanking LTR5_Hs

Chr	Position	Region	Structure	Length	*Preintegratioin* ^a^	Mismatches^b^	Reference^c^
chr3	125689444–125689493	3q21.2	solo_LTR	18	n.d.	2	this study
chr4	16078281–16079255	4p15.32	solo LTR^d^	96	n.d.	3	[13]
chr4	161582077–161582438	4q32.1	provirus	206	n.d.	1	this study
chr6	3055034–3055749	6p25.2	truncated provirus	111	determined	1	this study
chr7	16237347–16238314	7p21.2	solo_LTR	250	determined	2	[13]
chr13	23387789–23388759	13q12.12	solo_LTR	13	n.d.	2	this study
chr16	74833302–74834258	16q23.1	solo_LTR	61	n.d.	0	[13]
chr21	19933659–19941962	21q21.1	provirus	450	n.d.	0	this study

^a^ *n.d.* Not determined

^b^Number of mismatches and gaps between the duplicated sequences on the left and right

^c^Previously reported by Mamedov et al. [13]

^d^Two nucleotides present between the estimated sequences and either of the LTRs

### Long TSD sequence at the 6p25.2 provirus site

The non-human-specific insertional polymorphism at 6p25.2 was also validated by genomic PCR. The TSD sequence measured 111 bp in length, which is one of the longest TSD sequences according to our estimates (Table 2 and Additional file 5: Figure S4), where it was 99.1% and 100% identical to the LTR-flanking duplicated sequences (Fig. 3a). The HML-2 locus at 6p25.2 has been assigned as the structure of one LTR and a deleted ORF according to RepeatMasker, but the 7-bp sequence flanking the deleted ORF region, “CCCAAGG” has not been annotated previously. The 7-bp sequence could be found in most of the HML-2-related ORFs, so we interpreted this short sequence as a remnant of contiguous ORFs. Unexpectedly, the HML-2 site at 6p25.2 is not human-specific and the orthologs are found in other primate genomes: chromosome 6 in chimpanzee panTro4 and orangutan ponAbe2, where the ERV ortholog and the flanking TSDs share high identity with HML-2 and the TSDs at 6p25.2 in the human genome (chimpanzee, 97.6%; orangutan, 96.7%) (Fig. 3b). However, no ERV ortholog was found in the corresponding regions of chromosome 8 in gibbon nomLeu3 and chromosome 4 in rhesus macaque rheMac3. In the corresponding regions, the sequences of gibbon and rhesus macaque share 88.5% and 88.6% nucleotide identities with the human genome sequence, respectively (Fig. 3c). These cross-species comparisons indicate that the HML-2 site in 6p25.2 originated from the integration of a retroviral element into the genome of an ancestral species before the divergence of Homininae and Ponginae.

## Discussion

In this study, DGV data were compared with RepeatMasker data to identify HML-2_pre sites. In addition to the six HML-2_pre sites extracted from the DGV archive (Table 1), we identified eight other insertionally polymorphic sites that had been assigned previously as HML-2_pre sites. We also found seven putative HML-2_pre sites, including the 6q14.1 provirus site (chr6: 78,426,662–78,436,083) (Additional file 1: Table S5). In these sites, the deleted regions were slightly longer or shorter than the theoretically estimated lengths and they did not strictly comply with the definition of insertional polymorphisms. Genomic PCR was performed for four of these sites but deletion variants were not detected. However, the possibility that these sites are also insertional polymorphisms cannot be excluded completely because in some of these HML-2 sites, loss of the proviral allele was inferred by the k-mer mapping method using the WGS sequence data from 2506 samples [3]. Loss of the proviral alleles could have been caused by genomic rearrangement. Therefore, further analyses are required to determine whether preintegration sequences are actually present in these sites in human populations.

The two HML-2 sites on chromosomes 7 and 6 investigated in this study clearly had long TSDs with lengths of 250 and 111 bp, respectively. The former long TSD was detected by Mamedov et al. using a library of human-specific LTRs, but the preintegration site was not detected in their two human samples [13]. The frequency of the preintegration allele at 7p21.2 was 0.5 according to our results obtained using 24 human samples (Table 1), so the preintegration allele might have been detected if more samples were used in the previous study. We inferred that the longest TSD among all the HML-2 sites was located in the 21q21.1 HML-2_provirus (chr21: 19,933,659–19,933,915), where the putative TSD measured 450 bp in length (Table 2 and Additional file 5: Figure S4). The 21q21.1 HML-2_provirus is assigned to the Denisova genome [14], but it is not present in the chimpanzee and orangutan reference genomes. We could not detect the preintegration site for the 21q21.1 HML-2 provirus in our test population (*n* = 48, data not shown) in this study or in a previous study [3]. Therefore, the HML-2 locus at 21q21.1 may be fixed in the human population. It is possible that the putative long TSDs shown in Table 2 are due to successive genomic rearrangements and they are not bona fide TSDs (Additional file 6: Figure S5). Following proviral integration within two segmentally duplicated regions and subsequent homologous recombination between LTRs, homologous recombination between the two segmentally duplicated regions could have occurred in the ancestral genome. This type of genomic rearrangement may have yielded long homologous sequences flanking a solo LTR. Thus, the duplicated sequences flanking the solo LTR could be assigned as “pseudo-TSDs” and a plausible preintegration site may also have been generated via homologous recombination. This model may also apply to the putative long TSDs of the flanking proviruses. However, it seems unusual that the putative long TSD flanking the truncated provirus at 6p25.2 was maintained after the deletion of the 3′-LTR in this model. The truncation point of the provirus in the region corresponding to 6p25.2 is a common feature of humans, chimpanzees, gorillas, and orangutans. In other primates, i.e., rhesus macaques, gibbons, and baboons, no ERV insertion has been found in the corresponding region. In another model for the 6p25.2 site, proviral integration within either of the duplicated regions could have occurred in the ancestral genome, and the provirus and flanking region extending to the other duplicated region might have been deleted (Additional file 7: Figure S6).

The allele frequencies of the 4q27 HML-2 site in Table 1 and the previous study were 0.292 and 0.915, respectively, and thus they differed greatly [3]. In addition, the allele frequency of the 8p11.23 preintegration site was 0.813, which is unexpectedly high considering that the presence of preintegration sites has not been reported in this site. These discrepancies might be explained by bias when selecting the population in our study or in previous studies. Thus, further fine tuning of the parameters, e.g., to achieve lower coverage for the reads, might have been required in the previous analyses, especially for the novel preintegration sites identified in this study.

The chained alignment data for human and non-human primates in the UCSC archives and a search of the basic local alignment showed that the truncated provirus at 6p25.2 (chr6:3,055,034–3,055,749) was an ortholog of the ERVs in the chimpanzee (chr6: 3,051,682–3,057,596) and orangutan (chr6: 3,053,569–3,060,123) genomes, although it is not fixed in human populations (Fig. 3). A similar sequence to the preintegration site of the HML-2_provirus at 6p25.5 was found approximately 5.5-kbp downstream of the HML-2 site (chr6:2,999,182–2,999,383). However, based on a BLAT search, we confirmed that the PCR product sequence of the 6p25.2 preintegration site (accession number: LC154976) was aligned in the upstream area. Therefore, we consider that the sequenced PCR product must be from the HML-2 allele and not from a similar sequence downstream. The existence of ERV orthologs in the chimpanzee and orangutan genomes suggests that the insertionally polymorphic state is retained in modern humans, and that the insertional polymorphism at 6p25.2 can be explained by the model of ancestral polymorphism. However, it is unclear whether the ERV ortholog is fixed or not fixed in the chimpanzee and orangutan genomes. In the genomes of 10 Western chimpanzees published by the PanMap project (http://panmap.uchicago.edu), we could not detect any insertionally variable state in the ortholog [15]. Intriguingly, the presence of the 111-bp long TSDs flanking the HML-2_provirus at 6p25.2 suggests the occurrence of homologous recombination between the two long TSDs, thereby resulting in the preintegration state suggested by the “pseudo-TSD” model. The question of whether the pre-sequence has been retained or if it is the result of a homologous recombination event during the evolutionary process also applies to other insertional polymorphism sites with long TSDs [16]. It is still difficult to provide a completely satisfactory answer to this question, but comparison of primate genomes and human SNP information related to TSDs might be help to understand the history of HML-2 sites with long TSDs.

## Conclusions

We used PCR to identify several new HML-2 preintegration sites from the database of human structural variants. In addition, we clearly demonstrated the existence of HERV insertions flanked by unconventionally long TSDs, which measured 250 bp and 111 bp in length. This suggests that the length range for retroviral TSDs is larger than considered previously.

## Additional files

Additional file 1: Table S1. Insertional polymorphisms in HML-2; **Table S2.** Source information for DGV data; **Table S3.** Primer information; **Table S4.** Long putative TSD sequences flanking LTR5_Hs; **Table S5.** Putative sites for HML-2 insertional polymorphisms. (XLSX 27 kb)

Additional file 2: Figure S1. Insertional polymorphisms and regional indels in HML-2. Schemes are shown for insertional polymorphisms (A) and regional indels (B). (PDF 663 kb)

Additional file 3: Figure S2. LTR site in 11q13.4. UCSC Genome Browser display for chr11:71,875,418–71,876,385. Repetitive elements are shown as black and gray bars. Red and blue bars denote gain and loss regions, respectively. (PDF 154 kb)

Additional file 4: Figure S3. Scheme of the TSD analysis. In total, 451 putative TSDs were finally inferred. (PDF 345 kb)

Additional file 5: Figure S4. Estimation of the TSD lengths. HML-2 TSD sequences were inferred from the LTR-flanking duplicated sequences. In total, 451 HML-2 sites were estimated. The indicated genomic positions on chr6 and chr7 show the HML-2 insertional polymorphisms detected in this study. (PDF 75 kb)

Additional file 6: Figure S5. Model of pseudo-TSDs flanking a solo LTR**.** This model may explain the long homologous sequences flanking solo LTRs. In this model, a plausible preintegration site is formed via homologous recombination between genomic regions containing a solo LTR within two segmentally duplicated regions (locus A and locus B). This model may also apply to pseudo-TSDs flanking a provirus. Green and red areas are TSD sequences for proviral integration at locus A and locus B, respectively. (PDF 139 kb)

Additional file 7: Figure S6. Model of pseudo-TSDs flanking a truncated provirus. This model may explain the long homologous sequences flanking the truncated provirus at 6p25.2. In this model, a plausible preintegration site is formed as a result of successive genomic rearrangements. (PDF 44 kb)

## Abbreviations

BLAT: The Basic Local Alignment Search Tool (BLAST)-like alignment tool
DGV: Database of Genomic Variants
ERV: Endogenous retrovirus
HERV: Human endogenous retrovirus
HML-2: Human mouse mammary tumor virus like-2
LTR: Long terminal repeat
ORF: Open reading frame
sLTR: Solo LTR
SNP: Single nucleotide polymorphism
TSD: Target site duplication
