# Supplementary material for: Insertionally polymorphic sites of human endogenous retrovirus-K (HML-2) with long target site duplications
Source: BMC Genomics. 2017 Jun 27;18:487. doi: 10.1186/s12864-017-3872-6 (PMC5488345; doi:10.1186/s12864-017-3872-6)
Supplement: Supplementary file 2 — Insertional polymorphisms and regional indels in HML-2. Schemes are shown for insertional polymorphisms (A) and regional indels (B). (PDF 663 kb) [file 12864_2017_3872_MOESM2_ESM.pdf]

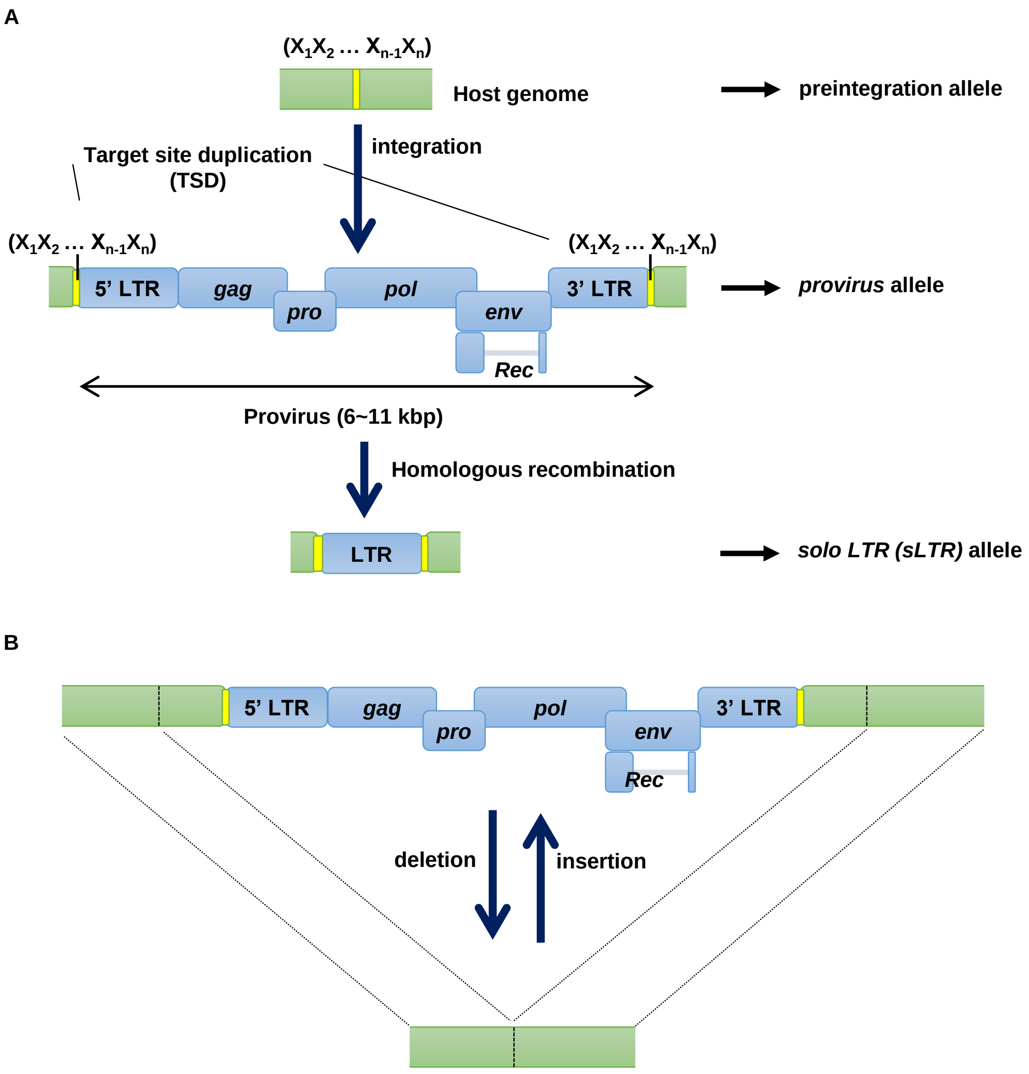

Additional file 2: Figure S1. Insertional polymorphisms and regional indels in HML-2. Schemes are shown fo[illegible] polymorphisms (A) and regional indels (B).
