# Supplementary material for: Insertionally polymorphic sites of human endogenous retrovirus-K (HML-2) with long target site duplications
Source: BMC Genomics. 2017 Jun 27;18:487. doi: 10.1186/s12864-017-3872-6 (PMC5488345; doi:10.1186/s12864-017-3872-6)
Supplement: Supplementary file 3 — LTR site in 11q13.4. UCSC Genome Browser display for chr11:71,875,418–71,876,385. Repetitive elements are shown as black and gray bars. Red and blue bars denote gain and loss regions, respectively. (PDF 154 kb) [file 12864_2017_3872_MOESM3_ESM.pdf]

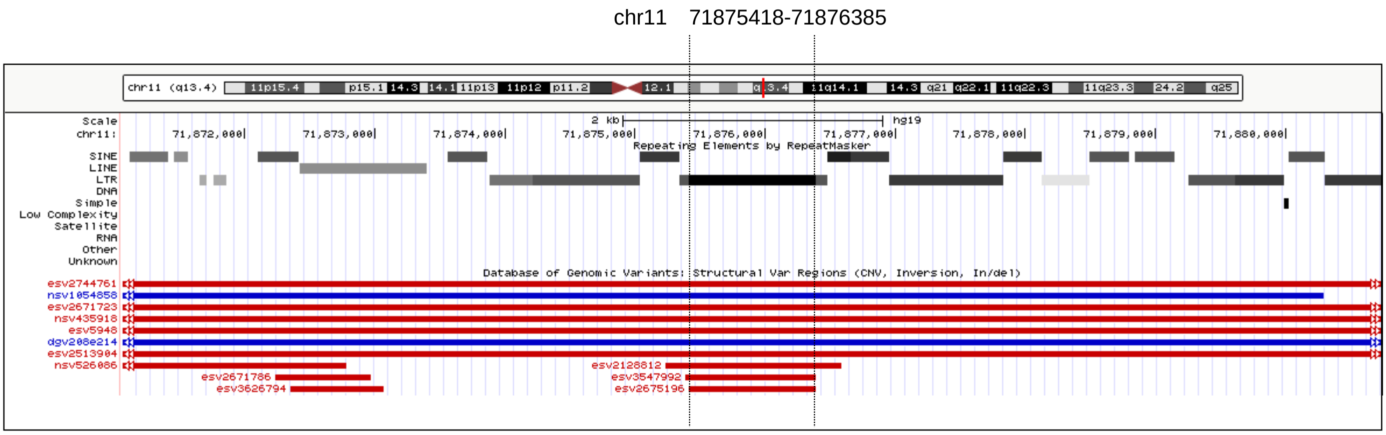

Additional file 3: Figure S2. LTR site in 11q13.4. UCSC Genome Browser display for chr11:71875418–71876385

elements are shown as black and gray bars. Red and blue bars denote gain and loss regions, respectively.
