# Supplementary material for: Insertionally polymorphic sites of human endogenous retrovirus-K (HML-2) with long target site duplications
Source: BMC Genomics. 2017 Jun 27;18:487. doi: 10.1186/s12864-017-3872-6 (PMC5488345; doi:10.1186/s12864-017-3872-6)
Supplement: Supplementary file 4 — Scheme of the TSD analysis. In total, 451 putative TSDs were finally inferred. (PDF 345 kb) [file 12864_2017_3872_MOESM4_ESM.pdf]

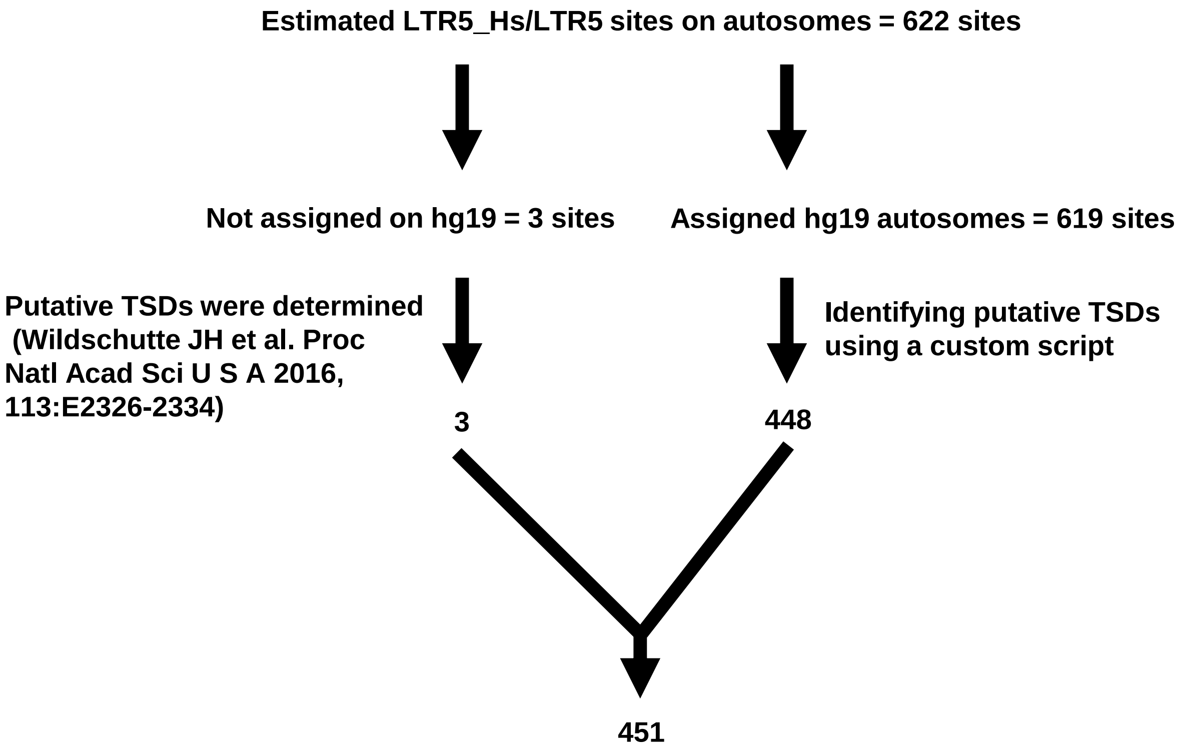

Additional file 4: Figure S3. Scheme of the TSD analysis. In total, 451 putative TSDs were finally inferred.
