# Supplementary material for: Insertionally polymorphic sites of human endogenous retrovirus-K (HML-2) with long target site duplications
Source: BMC Genomics. 2017 Jun 27;18:487. doi: 10.1186/s12864-017-3872-6 (PMC5488345; doi:10.1186/s12864-017-3872-6)
Supplement: Supplementary file 5 — Estimation of the TSD lengths. HML-2 TSD sequences were inferred from the LTR-flanking duplicated sequences. In total, 451 HML-2 sites were estimated. The indicated genomic positions on chr6 and chr7 show the HML-2 insertional polymorphisms detected in this study. (PDF 75 kb) [file 12864_2017_3872_MOESM5_ESM.pdf]

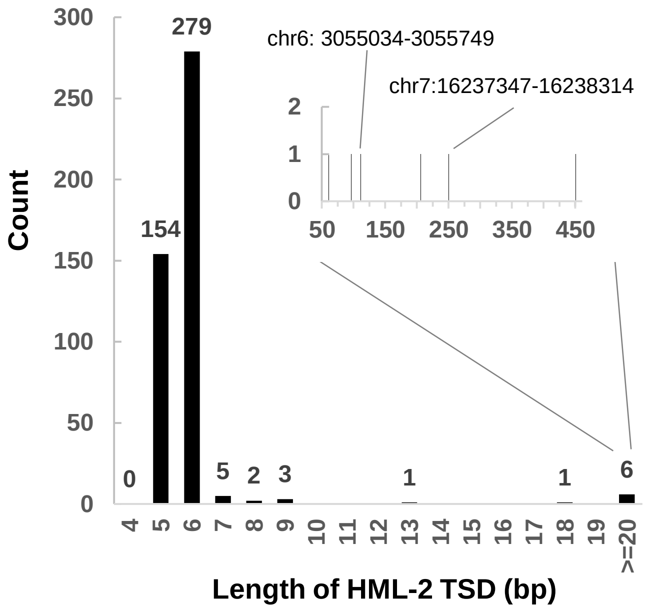

Additional file 5: Figure S4. Estimation of the TSD lengths. HML-2 TSD sequences were inferred from the LT[illegible] duplicated sequences. In total, 451 HML-2 sites were estimated. The indicated genomic positions on chr6 and chr7 show the HML-2 insertional polymorphisms detected in this study.
