# Supplementary material for: Insertionally polymorphic sites of human endogenous retrovirus-K (HML-2) with long target site duplications
Source: BMC Genomics. 2017 Jun 27;18:487. doi: 10.1186/s12864-017-3872-6 (PMC5488345; doi:10.1186/s12864-017-3872-6)
Supplement: Supplementary file 6 — Model of pseudo-TSDs flanking a solo LTR. This model may explain the long homologous sequences flanking solo LTRs. In this model, a plausible preintegration site is formed via homologous recombination between genomic regions containing a solo LTR within two segmentally duplicated regions (locus A and locus B). This model may also apply to pseudo-TSDs flanking a provirus. Green and red areas are TSD sequences for proviral integration at locus A and locus B, respectively. (PDF 139 kb) [file 12864_2017_3872_MOESM6_ESM.pdf]

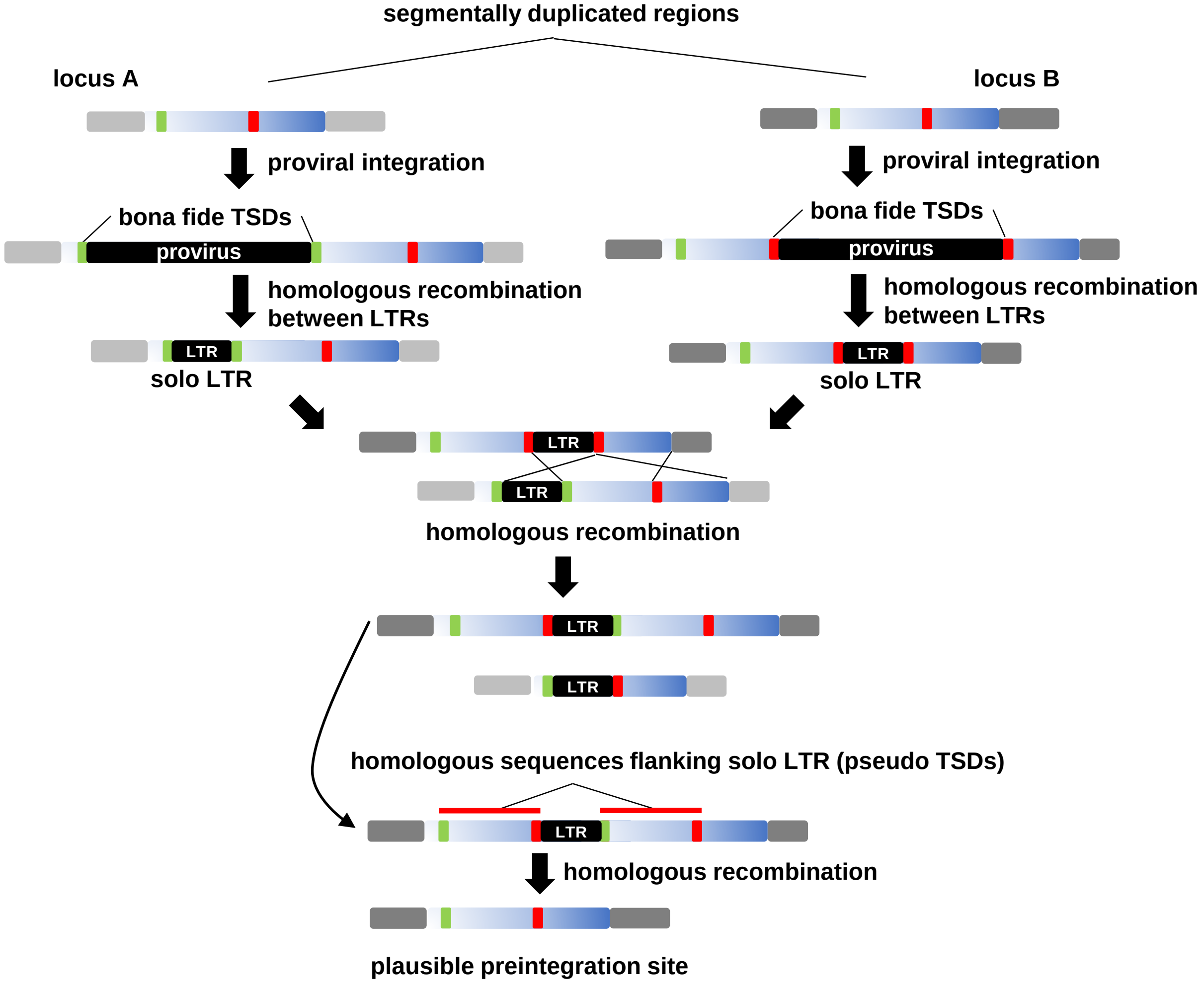

Additional file 6: Figure S5. Model of pseudo-TSDs flanking a solo LTR. This model may explain the long homologous sequences flanking solo LTRs. In this model, a plausible preintegration site is formed via homologous recombination between genomic regions containing a solo LTR within two segmentally duplicated regions (locus A and locus B). This model may also apply to pseudo-TSDs flanking a provirus. Green and red areas are TSD sequences for proviral integration at locus A and locus B, respectively.
