# Supplementary material for: Insertionally polymorphic sites of human endogenous retrovirus-K (HML-2) with long target site duplications
Source: BMC Genomics. 2017 Jun 27;18:487. doi: 10.1186/s12864-017-3872-6 (PMC5488345; doi:10.1186/s12864-017-3872-6)
Supplement: Supplementary file 7 — Model of pseudo-TSDs flanking a truncated provirus. This model may explain the long homologous sequences flanking the truncated provirus at 6p25.2. In this model, a plausible preintegration site is formed as a result of successive genomic rearrangements. (PDF 44 kb) [file 12864_2017_3872_MOESM7_ESM.pdf]

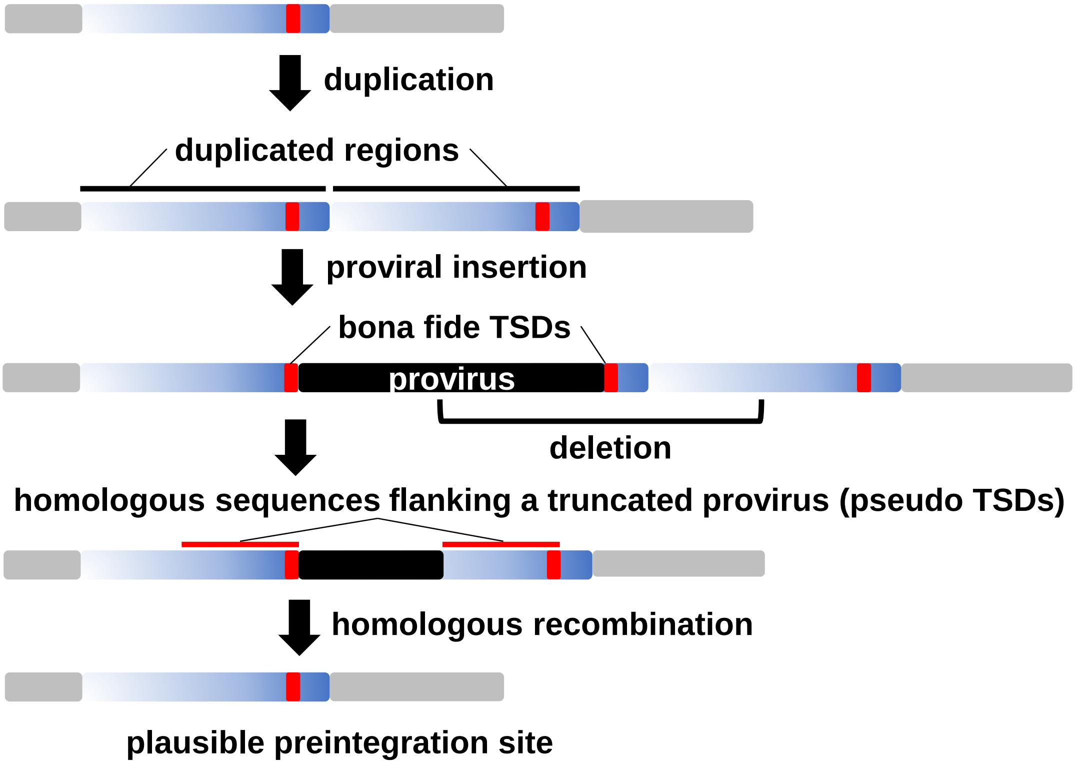

Additional file 7: Figure S6. Model of pseudo-TSDs flanking a truncated provirus. This model may explain the long homologous sequences flanking the truncated provirus at 6p25.2. In this model, a plausible preintegration site is formed as a result of successive genomic rearrangements.
